# Chondroid Syringoma: A Case Report

**Published:** 2015-06-11

**Authors:** Ranya Abi-Falah, Abel Gebre-Giorgis, Jennifer Rhodes, Christine Fuller

**Affiliations:** Division of Plastic and Reconstructive Surgery, Department of Surgery, Virginia Commonwealth University Health Center, Richmond, VA

**Keywords:** chondroid syringoma, mixed tumor, sweat glands, skin tumor, scalp

## DESCRIPTION

A 51-year-old man presented with a 1-year history of an enlarging mass on his right parietal scalp associated with symptoms of pain and tightness. The lesion was yellow and solid, with defined borders. There was no necrosis or ulceration. Excisional biopsy was performed and the surgical pathology report confirmed the diagnosis of chondroid syringoma.

## QUESTIONS

1. **What is a chondroid syringoma?**
2. **How to identify chondroid syringoma histologically?**
3. **Do benign lesions predispose to malignancy?**
4. **What is the recommended diagnostic and treatment modality for such lesions?**

## DISCUSSION

In 1859, Theodor Billroth first described chondroid syringoma as “an entity having the same histopathologic properties of mixed tumors of the salivary glands.”^1^ However, it was not until 1961 that Hirsch and Helwig coined the term “chondroid syringoma,” aptly named because of the histologic appearance of sweat gland features in a cartilage-like stroma.^2^ Chondroid syringoma is derived from epithelial and mesenchymal cells and comprises glandular elements of eccrine or apocrine type.^3^ Hirsch and Helwig defined the following histologic criteria for the characterization of chondroid syringoma: (1) nests of cuboidal or polygonal cells; (2) intercommunicating tubuloalveolar structures lined with 2 or more rows of cuboidal cells; (3) ductal structures composed of 1 or 2 rows of cuboidal cells; (4) occasional keratinous cysts; and (5) a matrix of varying composition. Chondroid syringoma may exhibit all 5 characteristics or manifest only some, with the most common feature being the nests of cuboidal or polygonal cells.

Chondroid syringoma are mostly benign entities that usually present asymptomatically in middle-aged men with a predilection for the head and neck region.^4^ The gross appearance is typically described as a slow-growing, solitary, nonulcerating mass ranging in size from 0.5 to 3.0 cm.^5^ However, cases of benign chondroid syringoma larger than 3.0 cm have been reported.^2^ Tumors larger than 3.0 cm are associated with a greater likelihood of malignancy.^5^ As of 2013, 30 cases of malignant chondroid syringoma have been described.^6^ Malignancy is more common in females, with no age predilection, and are observed more commonly on the extremities. Malignant chondroid syringoma typically arise de novo and not from a preexisting benign chondroid syringoma.^5^ Histologic features that suggest malignancy include cytologic atypia, tumor necrosis, numerous mitoses, excessive mucoid matrix, and poorly differentiated chondroid components.^6^

The diagnosis of chondroid syringoma is confirmed after histologic examination of tissue obtained by excisional biopsy. However, if presentation is questionable, a fine-needle aspiration may be of value since chondroid syringoma has been distinguished using this technique.^7^ Fine-needle aspiration has its limitations, such as sampling errors for histologic analysis that will require an experienced cytologist.^7^ The definitive approach for diagnosis and treatment is excisional biopsy. The surgeon needs to ensure that the margins are free of tumor. Regular follow-up is recommended to evaluate for recurrence following tumor excision, especially in the absence of negative margins.^7^ Review of the literature has shown a paucity of information regarding long-term follow-up after excision and recurrences. There is no known recurrence reported after complete excision of the lesion, with a short-term follow-up of 2 years.^7^

Chondroid syringoma is a benign, mixed-skin appendageal tumor of the sweat glands. It is rare, occurring in less than 0.1% of all excised skin lesions.^2^ It is derived from epithelial and mesenchymal cells, with glands of apocrine or eccrine type.^2^ Patients typically present with a slow-growing, solitary, nonulcerating subcutaneous nodule that has a predilection for the head and neck region.^4^ Diagnosis is confirmed with a histologic sample of the mass composed of nests of cells and ducts surrounded by chondromyxoid stroma.^8^ Treatment is achieved by surgical excision of the tumor with negative margins.^2^

## Figures and Tables

**Figure 1:**
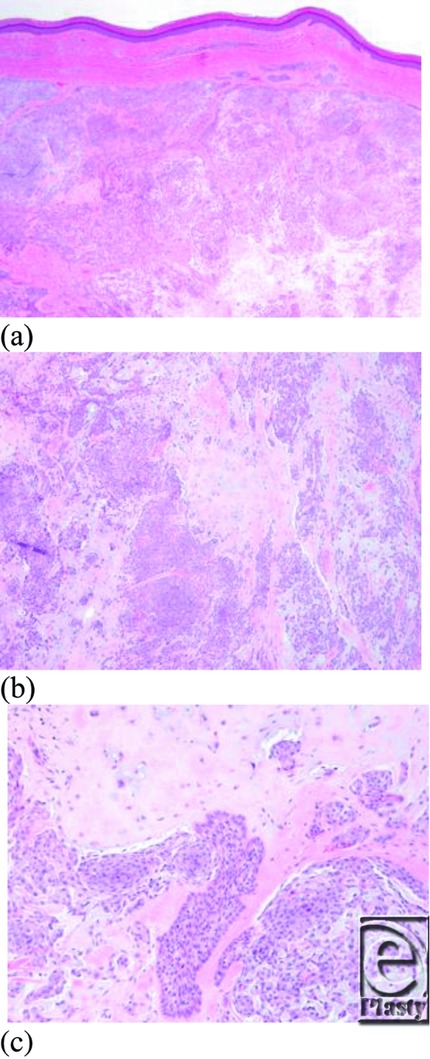
(a) Low-power (low 20×) photomicrograph of chondroid syringoma arising in the deep dermis with well-circumscribed appearance. (b) Medium (40×) and (c) high-power (100×) views show abundant chondromyxoid stroma enveloping islands and anastomosing cords of bland epithelial cells. (Hematoxylin and eosin, × 20, × 40, and × 100, respectively.)

## References

[1] Di Mare G, Vassallo L, Voglino C (2014). Chondroid syringoma: report of a case with uncommon location. J Med Diagn Methods.

[2] Yavuzer R, Başterzi Y, Sari A, Bir F, Sezer C (2003). Chondroid syringoma: a diagnosis more frequent than expected. Dermatol Surg.

[3] Hirsch P, Helwig EB (1961). Chondroid syringoma: mixed tumor of skin, salivary gland type. Arch Dermatol.

[4] Chen AH, Moreano EH, Houston B, Funk FG (1996). Chondroid syringoma of the head and neck: clinical management and literature review. Ear Nose Throat J.

[5] Sungur N, Uysal A, Gumus M, Kocer U (2003). An unusual chondroid syringoma. Dermatol Surg.

[6] Saxena A, Kamath N, Malik R (2013). A rare case of malignant chondroid syringoma of scalp. Indian Dermatol Online J.

[7] Skoro M, Ostovic KT, Cikara I, Muller D, Novak NP, Virag M (2010). Fine needle aspiration cytology of chondroid syringoma. Coll Antropol.

[8] Nasit J, Dhruva G (2012). Chondroid syringoma: a diagnosis by fine needle aspiration cytology. J Cutan Aesthet Surg.

